# Indications to respiratory syncytial virus immunoprophylaxis in the 29–32 wGA group: is there still room for debating?

**DOI:** 10.1186/s13052-017-0341-4

**Published:** 2017-02-02

**Authors:** Gianvincenzo Zuccotti, Valentina Fabiano

**Affiliations:** Department of Pediatrics, V. Buzzi Children’s Hospital, Università degli Studi di Milano, via Castelvetro 32, Milan, 20154 Italy

**Keywords:** Palivizumab, Respiratory syncytial virus, Prophilaxys, Preterm

## Abstract

Guidelines on immunoprophylaxis for prevention of RSV infection recommend it in preterm babies born before 29 wGA; in babies affected by bronchopulmonary dysplasia or congenital heart defects; and in post-heart transplantation patients. On the contrary, immunoprophylaxis is not recommended in preterm babies born between 29 and 35 wGA. We evaluated the impact of RSV-related healthcare expenditures in infants in the first 3 years of life in Italy, Lombardy Region. In light of the collected data and considering the cost of a complete palivizumab prophylaxis, extending it to babies 29–32 wGA, aged less than 6 months, appears to be a cost-effective strategy.

## Dear editor

It is well known that community-acquired respiratory syncytial virus (RSV) infection represents a significant healthcare issue in infants in the very first months of life, and in particular in those born prematurely.

A recent US observational cohort study [1] demonstrated that preterm infants born 29 to 35 weeks gestational age (wGA) not receiving immunoprophylaxis with palivizumab are at high risk for severe RSV disease, particularly in the first months of life. RSV disease represents also a risk factor for development of wheezing and asthma, reduced lung function, and irreversible airway obstruction [2]. Moreover, hospitalizations, Emergency Room and outpatient visits for community-acquired RSV disease are associated with significant resources consumption and increased healthcare costs, as demonstrated by a recently published Italian study [3].

Current Italian Guidelines recommend with a Level of Evidence II and Strength of recommendation A that: for infants of 29–35 weeks gestational age and age ≤6 months at the beginning of the epidemic season, prophylaxis with palivizumab might be taken into consideration in presence of risk conditions predisposing to severe infections and/or need for hospitalization [4, 5]. On the contrary, the recent recommendations of the American Academy of Pediatrics, removed otherwise healthy preterm infants born > 29 weeks of gestation from the high-risk groups recommended to receive palivizumab prophylaxis. Consequently, the Italian Drug Agency (AIFA) most recently decided the total financial coverage by the National Health Service of the palivizumab prescription to the healthy preterms only for the <29 wGA group and age ≤12 months.

We evaluated the total number of hospitalizations and Emergency Room and/or outpatient visits in infants in the first 3 years of life in Italy, Lombardy Region, and the related total expenditures by the Regional Healthcare System. We considered 65.496 babies born alive in 2013, classified by sex and by GA in four groups: 24–28 wGA, 29–32 wGA, 33–34 wGA, ≥ 35 wGA (Table 1). For all groups, the total number of hospitalizations in the period 2013–2015 was 22.974. 680 babies in the 29–32 wGA group needed 731 hospitalizations and 600 babies in the same group needed 11.603 Emergency Room and/or outpatients visits; with a total expenditure of 2.121.775 € (median expenditure per patient: 3120 €) for hospitalizations, and of 583.996 € for other healthcare facilities use. We analyzed the hospitalizations according to the International Classification of Diseases 9^th^ Revision (ICD-9), considering the codes 079.6, corresponding to RSV infection; 446.x corresponding to acute bronchitis, acute bronchitis caused by RSV, acute bronchiolitis caused by viruses other than RSV; 485 pneumonia; and 486 corresponding to pneumonia caused by unspecified agents. 16.8% (3616) of hospitalizations was performed because of diseases included in one of these ICD-9 codes; most frequent diagnoses were those included in the 466.x ICD-9 code, reported in 2764 (12.9%) hospitalizations. According to GA, 93 babies of the 29–32 wGA group were hospitalized for one of these diagnoses, and 73 of these for acute bronchitis, acute bronchitis caused by SRV, acute bronchiolitis caused by viruses other than RSV (ICD-9 code 466.x).

**Table 1:** Distribution by sex and gestational age of babies born alive in 2013 in Lombardy Region

Gestational Age (weeks)	Male		Female		Total	
	*n*°	%	*n*°	%	*n*°	%
24–28	124	0,4	101	0,3	225	0,3
29–32	328	1,0	352	1,1	680	1,0
33–34	575	1,7	507	1,6	1.082	1,7
> = 35	32.681	96,9	30.828	97	63.509	97
Total	33.708	100	31.788	100	65.496	100

The cost of a complete palivizumab prophylaxis (5 doses) is 4071 € per patient. If we had performed palivizumab immunoprophylaxis in the 680 babies 29–32 wGA who were hospitalized, total expenditure would have been 2,8 million €. The total cost of the 93 hospitalizations in the 29–32 wGA group for three years was 1,6 million €, and the cost of other healthcare facilities consumption was 0,6 million €, giving a total expenditure of 2,2 million €.

If we consider to administer palivizumab prophylaxis in babies 29–32 wGA and aged less than 6 months (presumably 70% of them), we may estimate a total expenditure of 1,9 million €, saving more or less 0,3 million €. In addition, if we consider also the disease impact on chronic respiratory sequelae and the many other disease-related indirect costs, we may suppose that healthcare costs saving would be even greater.

In light of these data, extending palivizumab prophylaxis to babies 29–32 wGA, aged less than 6 months, appears to be a cost-effective strategy. Because of the limitations of the study to a single Italian region, these findings cannot be generalized, but strongly point to a need to reevaluate the role of palivizumab prophylaxis in the 29–32 wGA subpopulation. Debate on RSV prophylaxis is maybe not still definitely closed.
